# The impact of building height on urban thermal environment in summer: A case study of Chinese megacities

**DOI:** 10.1371/journal.pone.0247786

**Published:** 2021-04-22

**Authors:** Meiya Wang, Hanqiu Xu

**Affiliations:** 1 School of History and Geography, Minnan Normal University, Zhangzhou, Fujian, China; 2 College of Environment and Resources, Institute of Remote Sensing Information Engineering, Fujian Provincial Key Laboratory of Remote Sensing of Soil Erosion and Disaster Prevention, Fuzhou University, Fuzhou, Fujian, China; Northeastern University (Shenyang China), CHINA

## Abstract

The quantitative relationship between the spatial variation of building’s height and the associated land surface temperature (LST) change in six Chinese megacities is investigated in this paper. The six cities involved are Beijing, Shanghai, Tianjin, Chongqing, Guangzhou, and Shenzhen. Based on both remote sensing and building footprint data, we retrieved the LST using a single-channel (SC) algorithm and evaluate the heating/cooling effect caused by building-height difference via correlation analysis. The results show that the spatial distribution of high-rise buildings is mainly concentrated in the center business districts, riverside zones, and newly built-up areas of the six megacities. In the urban area, the number and the floor-area ratio of high to super high-rise buildings (>24m) account for over 5% and 4.74%, respectively. Being highly urbanized cities, most of urban areas in the six megacities are associated with high LST. Ninety-nine percent of the city areas of Shanghai, Beijing, Chongqing, Guangzhou, Shenzhen, and Tianjin are covered by the LST in the range of 30.2~67.8°C, 34.8~50.4°C, 25.3~48.3°C, 29.9~47.2°C, 27.4~43.4°C, and 33.0~48.0°C, respectively. Building’s height and LST have a negative logarithmic correlation with the correlation coefficients ranging from -0.701 to -0.853. In the building’s height within range of 0~66m, the LST will decrease significantly with the increase of building’s height. This indicates that the increase of building’s height will bring a significant cooling effect in this height range. When the building’s height exceeds 66m, its effect on LST will be greatly weakened. This is due to the influence of building shadows, local wind disturbances, and the layout of buildings.

## Introduction

The rapid development of cities has led to fast growth of urban space in China [1, 2]. In many large cities, high-rise buildings have been constructed to meet the demands of the rapid growth of urban population. Compared with low-rise buildings, high-rise buildings save more floor space and improve the efficiency of landuse, which can leave more space for the construction of urban ecological land. Nonetheless, high-rise buildings have a stronger influence on the local atmospheric conditions and sunshine conditions than low-rise buildings. It has further effects on local ecosystems, energy and water demands, human well-being [3, 4]. In order to explore the rationality of urban vertical expansion, it needs to investigate the relationship between building height and land surface temperature (LST).

Compared with traditional in situ data, remote sensing data could provide continuous and mass data, which is widely used to retrieve LST, and the distribution, dynamicity, and reveal heterogeneity of LST [5–7]. The different thermal infrared remote sensing data, such as MODIS and Landsat, have been used to demonstrate the heating/cooling effect of urban land cover change. Many studies have measured land surface temperature through single-window algorithm, single-channel algorithm, split window method, etc. [8–11].

The relationship between urban morphology factors on LST has been widely researched. Zhao et al. [12] studied the thermal effects of various building types day and night. Du et al. [13], Ng et al. [14] and Zhan et al. [15] studied the impact of building density on the urban thermal environments. Moreover, many scholars have focused on the relationships between the LST and land use, sky view factor, ventilation performance, building height and street pattern, in both the neighborhood and precinct scales [16–23]. The relationship between the urban thermal environment and different urban morphology factors varies in different cities due to differences in the climate zone, population, scales and methods [24, 25]. Guo et al. (2020) used urban spatial form indices, e.g., building form, landuse, landscape index, socio-economic, terrain, remote sensing index, to explore spatial and temporal differentiation characteristics and driving factors of LST at the community scale [20]. The results showed that LST was influenced by land use. Yang et al. (2019) assessed the impact of urban building morphology on local climate surface temperatures under different wind conditions [21]. The results showed that urban building morphology was one of the important drivers of climate change. High-density high-rise buildings can increase surface temperatures. He et al. (2019, 2020) comprehensively analyzed the influence of urban surface structures, including compactness, building height and street structure, on local ventilation performance and urban thermal environment [22, 23]. Result indicates the combinations of the open mid-rise gridiron precinct with different external meteorological conditions resulted in significant variations in precinct ventilation performance and urban thermal environment. Thus, it can be seen that the reasonable urban spatial form plays an important role in mitigating urban heat islands [23].

The spatial configuration of urban buildings is often considered when studying the influence of urban development on the urban thermal environment. The three-dimensional form of the urban buildings is an important factor reflecting the urban development status. Understanding the mechanisms behind the LST and its variability caused by three-dimensional change of buildings has practical significance. In conclusion, previous studies only focus on analyzing the degree of correlation relationship between urban morphology factors, e.g., building height and street pattern, and urban temperature. So far, little research has got their quantitative relationship. Accordingly, we aim to establish the quantitative function relationship of building height and land surface temperature in six China’s megacities and to explore the appropriate building height value from the perspective of the impact of building height on urban thermal environment. The height of the six megacities has a wider value range than others, which helps data comparison and the reliability of the research results. In this study, Landsat images were used to retrieve LST, which was then combined with the building footprints data to discover the relationship between building height and LST. We analyze the thermal environment effect resulting from the development of urban building height associated with rapid urbanization, and thus provide a theoretical basis for study of the improvement of thermal environment. It is helpful to address the practical problems associated with urban development and to improve the urban living environment.

## Study areas

**Fig 1:**
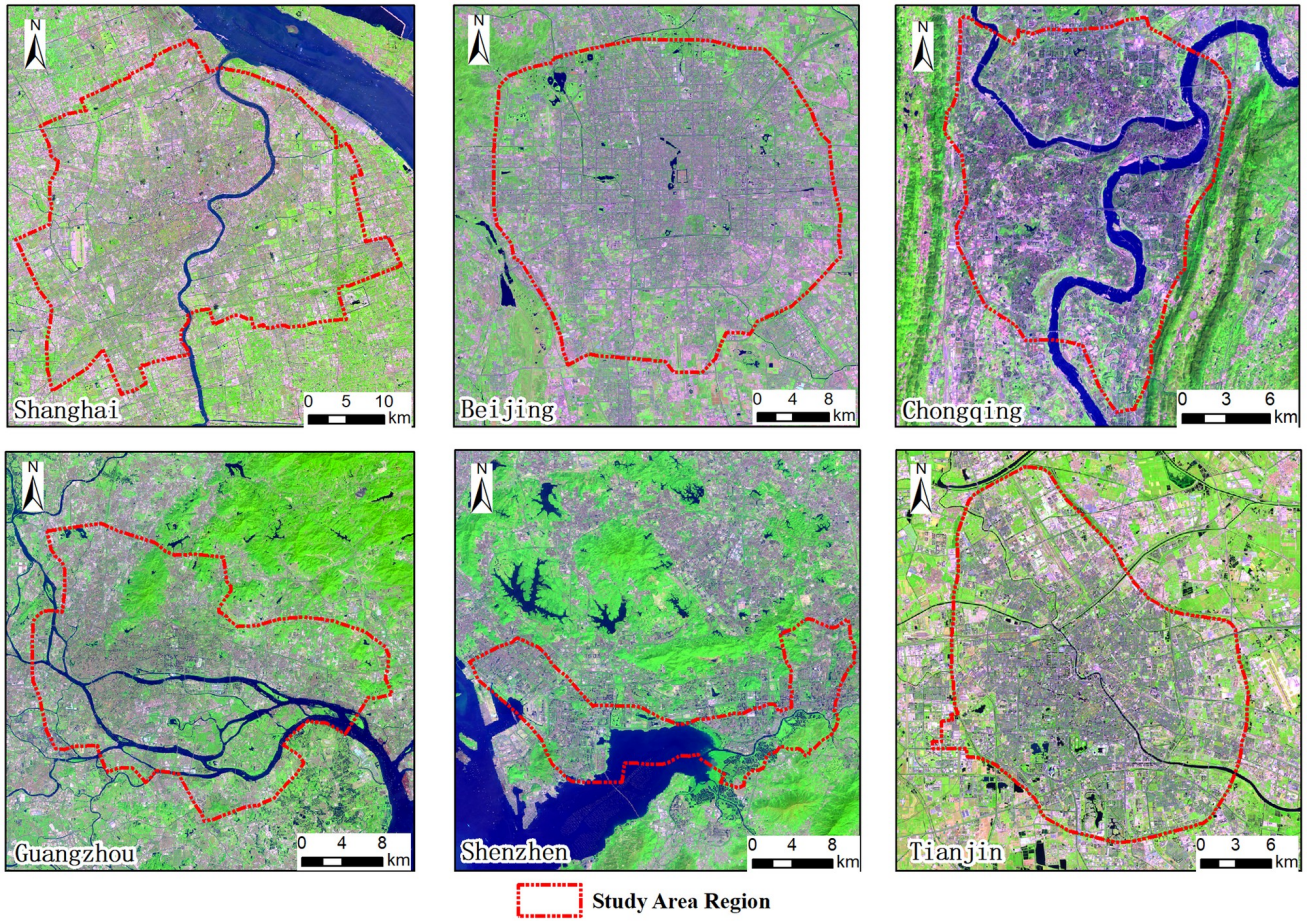
Landsat 8 OLI images of the six megacities (downloaded from USGS/NASA website).

According to the notice of the state council on adjusting the scale of urban scale in 2014 [26], the six megacities that have more than 10 million permanent population were selected as the study cities. The six megacities are Shanghai (24.24 million), Beijing (18.63 million), Chongqing (15.08 million), Guangzhou (13.15 million), Shenzhen (13.03 million) and Tianjin (12.97 million) by referring to the population data of China City Construction Statistical Yearbook in 2018 (Fig 1). Among them, Shanghai, Beijing, Chongqing, and Tianjin are municipalities of China. Guangzhou is the provincial capitals of Guangdong. Shenzhen is China’s first special economic zone established in 1978. It is also considered as one of the fastest growing cities in the world. Beijing (39°26’-41°03’N, 115°25’-117°30’E) and Tianjin (38°33’-40°15’N, 116°42’-118°04’E) are located in the North China Plain of north China. Shanghai (30°40′-31°53′N, 120°52′-122°12′E) is located in the Yangtze Delta Plain of east China. Guangzhou (22°26’-23°56’N, 112°57’-114°03’E) and Shenzhen (22°27’-22°52’N, 113°46’-114°37’E) are located in Pearl River Delta Plain of southeast China. Chongqing (28°10’-32°13’N, 105°11’-110°11’E) is located in the Sichuan basin of southwest China. These six megacities have large population with a lot of tall buildings. Studying on the relationship between building height and urban thermal environment in these megacities can reveal the relationship between urban construction and ecological environment after rapidly urbanization. Beijing’s 5th ring road, Shanghai’s outer ring highway, Guangzhou’s loop expressway, Chongqing’s inner ring expressway, Shenzhen’s Ring Avenue and Tianjin’s outer ring highway are respectively taken as the study area’s boundary. These boundaries can effectively extract the buildings inbuilt-up area and ensure the integrity of the building units.

## Methods

### Building height distribution

Building height means the height difference between the roof and the outdoor floor. We obtained the building footprints and their heights data in the urban area from Urban Data Institute (a Chinese institution shared for geographic information data) in the first quarter of 2018. By referring to the high spatial resolution Google earth imagery, we found most of the buildings were mapped in the building footprint dataset. The very few missing buildings were digitized by referring to the Google Earth imagery. The dataset also included the number of floors for each building. We further calculated the building height by multiplying the number of floors by 3 m, which is defined as the typical height of one floor by the Residential Building Code of China [27]. The building height data has also been corrected by referring to the online building height information of the six megacities and Google high resolution imagery. Finally, we used Arc Scene software to do 3D building modeling.

We classify building height into five types based on the number of floors of the buildings (Table 1), according to the Chinese building classification in the Uniform standard for Design of Civil Buildings [28].

**Table 1:** Classification of building types.

Building type	Storey
Low-rise building	1~3(3~10m)
Multi-rise building	4~6(10~18m)
Mid-rise building	7~9(18~24m)
High-rise building	>9(24~100m)
Super high-rise building	Building height exceeds 100m

### LST retrieval

Landsat 8 thermal infrared sensor (TIRS) data was used to calculate the LST. It is downloaded from the United States Geological Survey (USGS) (https://earthexplorer.usgs.gov/). The details of the data are shown in Table 2. The selected imagery all obtained on the similar relatively clear weather conditions in summer and the air temperatures were over 30°C. These images have a similar overpass time (during10:24–10:52 a.m. CST (China Standard Time)) so that the LSTs could be compared with each other.

**Table 2:** Landsat 8 TIRS images.

City	Date	Time (CST)	Spatial resolution (m)	Path/Row	Cloud cover of study area (%)
Shanghai	2018-7-26	10:24 a.m.	100	118/38, 118/39	5.50
Beijing	2018-6-27	10:52 a.m.	100	123/32	0.42
Chongqing	2018-9-02	11:26 a.m.	100	128/39	0.49
Guangzhou	2018-7-22	10:52 a.m.	100	122/44	0.05
Shenzhen	2018-7-22	10:52 a.m.	100	122/44	1.89
Tianjin	2018-7-06	10:46 a.m.	100	122/33	0.92

Landsat 8 official website recommends using band 10 to retrieve LST due to the calibration problem of band 11 [29, 30]. Therefore, Landsat 8 band 10 was used to retrieve the LST using the Single-channel method (SC) [3, 31]. The algorithm need less atmospheric parameter and can achieve a lower root mean square error [32, 33]. Firstly, at-sensor brightness temperatures (*T*_sensor_) were retrieved based on the atmospheric correction of TIRS 10 thermal bands. The formulas are expressed as:

$$L_{\text{sensor}}=M_{\text{L}}\cdot DN+A_{\text{L}}$$ (1)

$$T_{\text{sensor}}=K_{2}/\text{ln}(K_{1}/L_{\text{sensor}}+1)$$ (2)

Where *L*_sensor_ is the at-sensor spectral radiance of TIRS band 10; *M*_L_ and *A*_L_ are multiplicative re-scaled factor corresponding to a specific band and additive re-scaled factor corresponding to a specific band, respectively. *DN* is the calibrated standard product pixel value of band 10. *K*_1_ and *K*_2_ are the band-specific thermal conversion constants of band 10. LST was subsequently obtained after emissivity correction through Eqs (3) and (4):

$$LST=\gamma [\varepsilon^{-1}(\psi_{1}L_{\text{sensor}}+\psi_{2})+\psi_{3}]+\delta$$ (3)

$$\gamma \approx T/(b_{\gamma }L),\;\delta \approx T-T/b_{\gamma }$$ (4)

where *γ* and *δ* are two parameters dependent on the Planck’s function. *ε* is the surface emissivity, which can be estimated from the ASTER spectral database and the result of [34]. The emissivity of TIRS band 10 for forest, grass, soil, built land and water is 0.9813, 0.9823, 0.9722, 0.9212 and 0.9908, respectively. *ψ*_1_, *ψ*_2_, *ψ*_3_ are the atmospheric functions. *b*_γ_ = 1324 for TIRS band 10; *ψ*_1_, *ψ*_2_, *ψ*_3_ are the atmospheric functions.

### Building shade extraction

In order to analyze the effects of shadow created by high building to LST, automated Water Extraction Index (*AWEI*_sh_) [35] is used to effectively extract water and shadow pixels. Then, modified normalized difference water index (MNDWI) [36] is used to removing water pixels from shadow pixels. The formula is expressed as:

$$AWEI_{\text{sh}}=\rho_{\text{band}2}+2.5\times \rho_{\text{band}3}-1.5\times (\rho_{\text{band}5}+\rho_{\text{band}6})-0.25\times \rho_{\text{band}7}$$ (5)

$$MNDWI=(\rho_{\text{band}3}-\rho_{\text{band}6})/(\rho_{\text{band}3}+\rho_{\text{band}6})$$ (6)

where *ρ* is the reflectance value of spectral bands of Landsat 8: *band*2 (Blue), *band* 3 (Green), *band* 5 (NIR), *band* 6 (SWIR1) and *band* 7 (SWIR2).

### Correlation analysis

Our correlation analysis focused on how LST changed through building height. Bivariate correlation analysis is used primarily with two or more variables. Here, we employ the Pearson simple correlation coefficient to measure the linear correlations between building height and LST. The formula is expressed as:

$$r=\frac{\sum (x-\bar{x})(y-\bar{y})}{\sqrt{\sum {\left(x-\bar{x}\right)}^{2}\sum {\left(y-\bar{y}\right)}^{2}}}$$ (7)

where *r* is the correlation coefficient between *x* and *y*. The correlation coefficient must lie in the range 0≤r≤1. The two variables are positive correlated in the range *r*>0; the two variables are negatively correlated in the range *r*<0; the two variables have no linear correlation in the range *r* = 0; *x*, *y* mean the sample value. $\bar{x}$ and $\bar{y}$ are the mean values of *x* and *y*, respectively.

## Results

### High-rise buildings distribution

**Fig 2:**
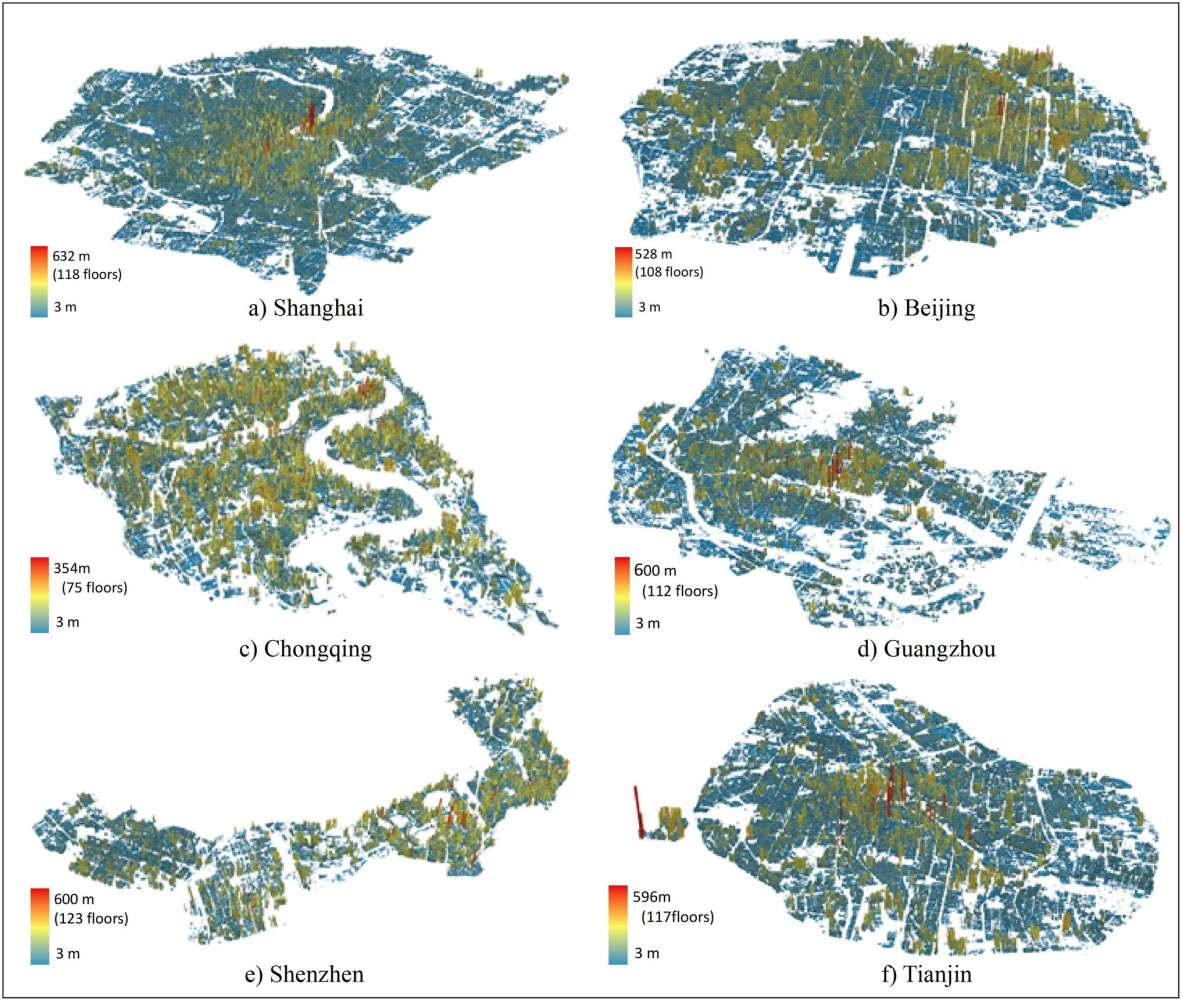
Three-dimensional map of building distribution.

**Fig 3:**
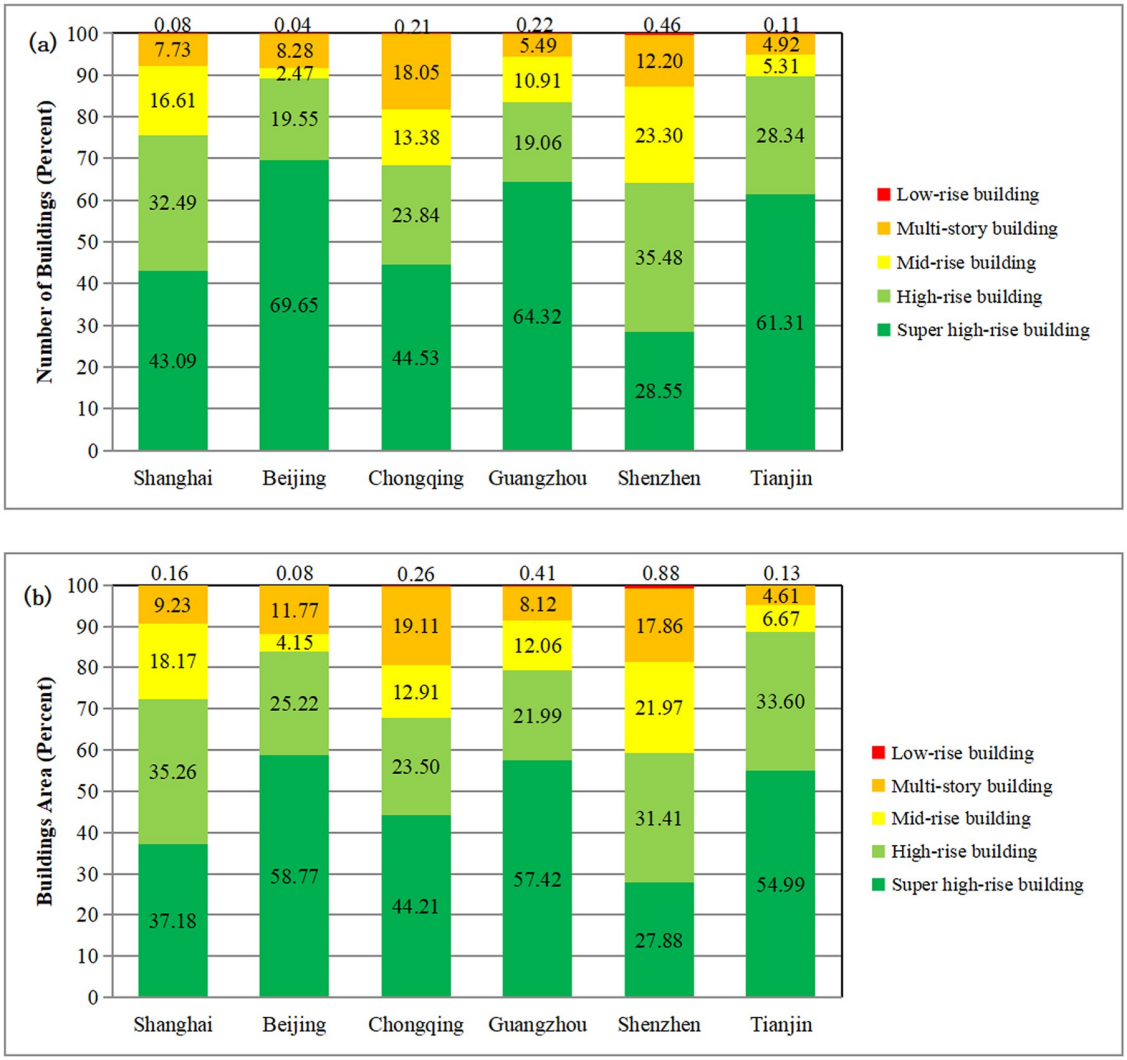
Histograms showing: Numbers percent of buildings according to height (a) and building area ratio (b).

The rapid urbanization leads to the rapid growth of urban 3D space. Building height is an important index to measure the vertical spatial distribution of buildings. We now consider the spatial distribution of high-rise buildings and super high-rise buildings were mainly concentrated in the urban Center Business Districts, Riverside zone, and new built-up area. The special regions are Shanghai’s Central Business Districts (Lujiazui and Xujiahui regions) (Fig 2a), Beijing‘s east and west part along the second and third ring roads (Fig 2b), Chongqing’s Central Business Districts (Jiefangbei, Jiangbeizui and Danzishi region) (Fig 2c), Guangzhou’s Central Business Districts (Zhujiang and Tianhe New Town) and the region along both sides of the Pearl River (Fig 2d), Shenzhen’s business center (Futian and Luohu region) (Fig 2e), Tianjin’s central urban areas (Heping, Hexi and Hebei region) and the region along the Haihe River (Fig 2f). In 2018, the number percent and building area ratio of high-rise buildings (24-100m) and super high-rise buildings (>100m) are all over 5%and 4.74% in the urban area of the six megacities, respectively. Among the six megacities, Chongqing’s urban area has the largest high-rise building number percent (18.26%) and building area ratio (19.37%) (Fig 3).

### Land surface temperature distribution

**Fig 4:**
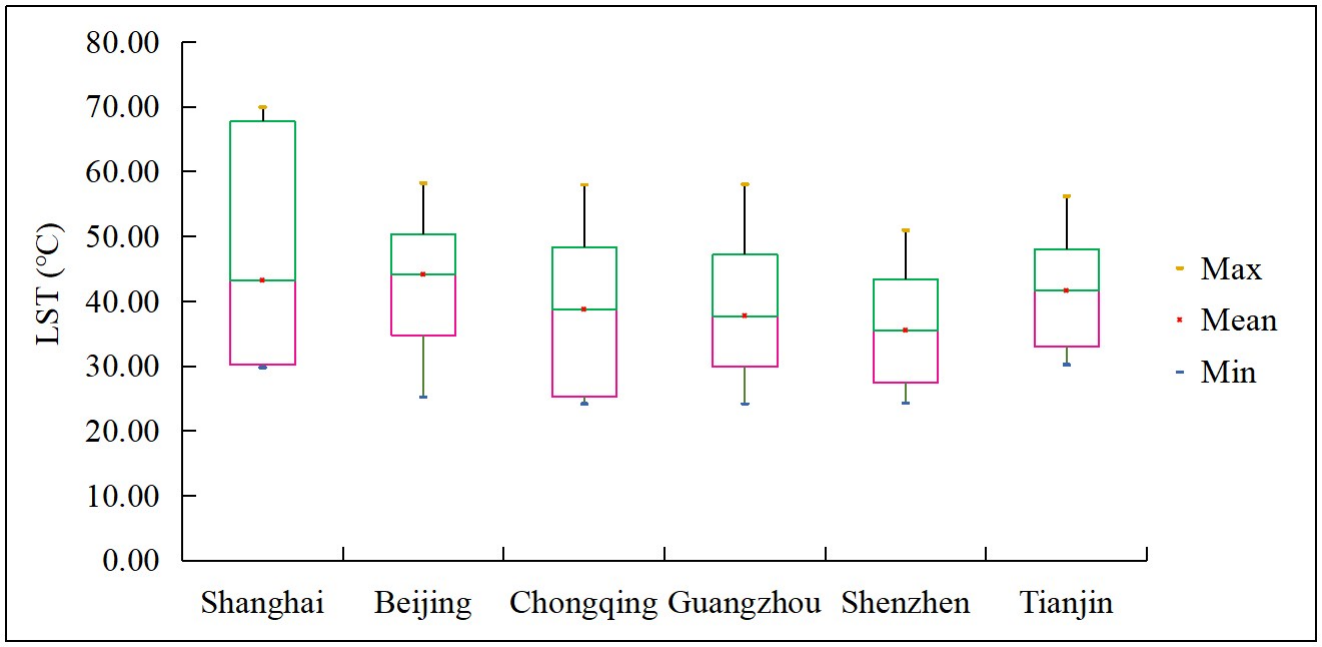
Box plots of LST distributions.

The LST results are shown in Fig 4. Due to the lack of the historical temperature data of the standard meteorological stations in the six megacities, we used MODIS LST data to verify the accuracy of L8 LST data [37]. The MODIS LST daily data (MOD11A1) was obtained by the National Aeronautics and Space Administration (NASA). The mean L8 LST results were compared to the mean MODIS LST data in the same region. By comparing the mean LST difference, it can avoid the error caused by the difference in spatial resolution of the two kinds of imagery. The experimental results show that the error between the retrieved Landsat LST and the MODIS LST is less than 5%, which can reflect the spatial distribution of the LST in the study area (Table 3). It is due to the difference in the overpass time of the two satellites.

**Table 3:** Results comparison of retrieve accuracy of LST.

City	Date	Overpass Time (CST)	Satellite	Mean LST(°C)	Mean LST Difference(°C)
Shanghai	2018/7/26	11:36a.m.	MODIS	36.22	-0.76
		10:24a.m.	Landsat	35.46	
Beijing	2018/6/27	11:48a.m.	MODIS	36.19	0.57
		10:52a.m.	Landsat	36.76	
Chongqing	2018/9/2	10:06a.m.	MODIS	37.04	1.23
		11:26a.m.	Landsat	38.27	
Guangzhou	2018/7/22	10:00a.m.	MODIS	34.34	0.84
		10:52a.m.	Landsat	35.18	
Shenzhen	2018/7/22	10:00a.m.	MODIS	34.26	0.97
		10:52a.m.	Landsat	35.23	
Tianjin	2018/7/6	11:42a.m.	MODIS	34.22	-0.68
		10:46a.m.	Landsat	33.54	

**Fig 5:**
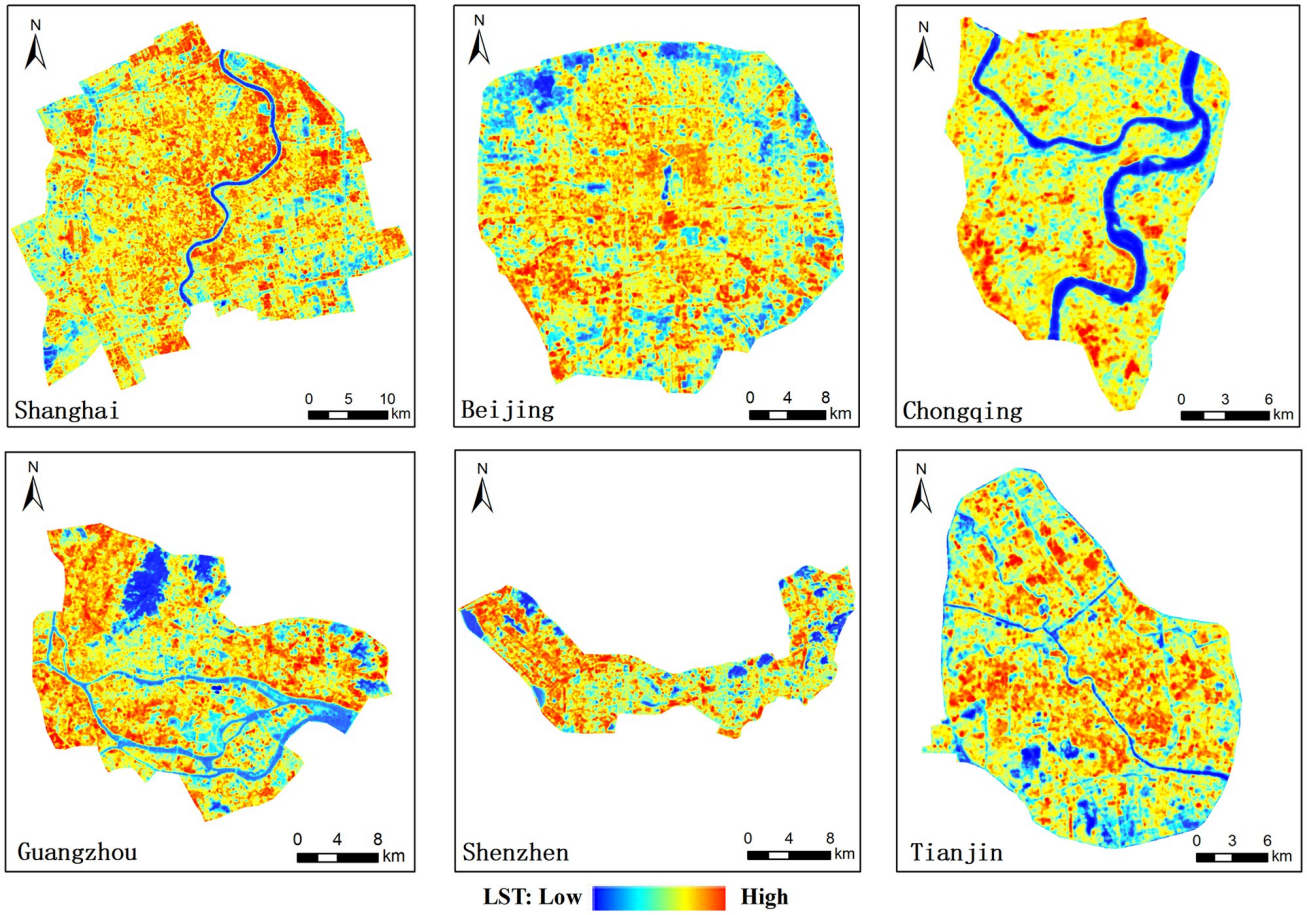
Spatial distribution of LST of six megacities (USGS/NASA Landsat 8).

We now consider the spatial distribution of LST (Figs 4 and 5). Most of urban area in the six megacities covered by high LST showed orange and red colors in Fig 5. In Shanghai, the LST in the north industrial area and old central is relatively high, and the LST in the outer new built-up area is relatively low (Fig 5a). 99% of grid cells had a LST within the range from 30.2 to 67.8°C (Fig 4). In Beijing, the LST in the old central (Dongcheng District, Xicheng District) and south Fengtai District is relatively high, and the LST in the north is relatively low (Fig 5b). 99% of grid cells had a LST within the range from 34.8 to 50.4°C (Fig 4). In Chongqing, the LST in the southwest Shixinlu industrial area, southeast Dashilu industrial area and Jiulongpo District is relatively high, and the LST in the north area along Jialing River is relatively low (Fig 5c). 99% of grid cells had a LST within the range from 25.3 to 48.3°C (Fig 4). In Guangzhou, the LST in the north Baiyun District, east Huangpu District and South industrial area is relatively high, and the LST in the central Yuexiu District is relatively low (Fig 5d). 99% of grid cells had a LST within the range from 29.9 to 47.2°C (Fig 4). In Shenzhen, the LST in the northwest Bao’an District is relatively high, and the LST in the central Nanshan District and west Futian District and Luohu District is relatively low (Fig 5e). 99% of grid cells had a LST within the range from 27.4 to 43.4°C (Fig 4). In Tianjin, the LST in the north Nankai District and Beichen District is relatively high, and the LST in the east and south is relatively low (Fig 5f). 99% of grid cells had a LST within the range from 33.0 to 48.0°C (Fig 4).

### Correlation of land surface temperature with building height

**Fig 6:**
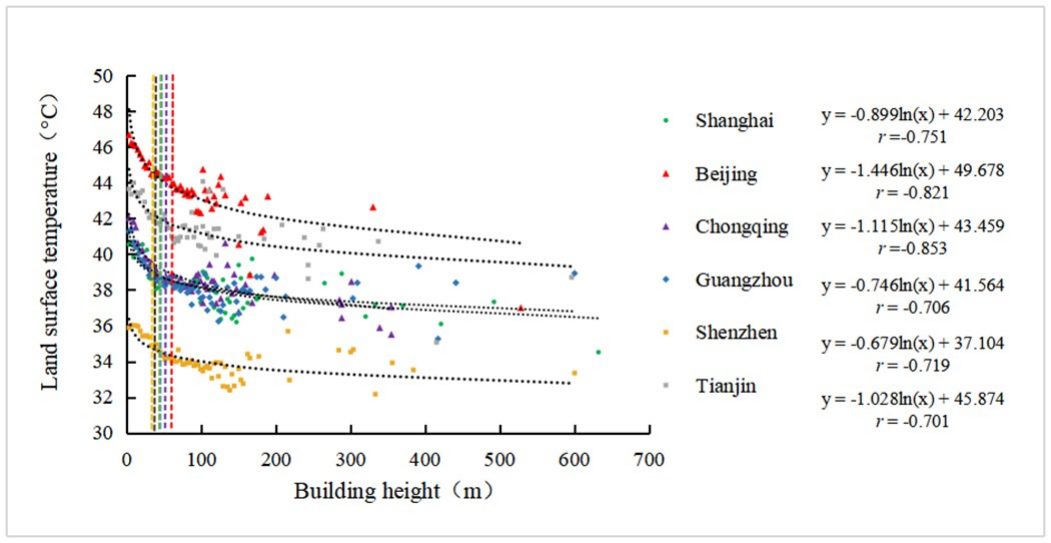
Regression analysis of building height and land surface temperature.

According to the regression analysis result (Fig 6), building height and LST has negative logarithmic correlation with the coefficient value from -0.701 to -0.853. It shows that LST decreases with the increase in building height. In other words, the area with low-rise buildings has higher LST, while the area with high-rise buildings has lower LST. Especially, within the height range of 0~66m, it will bring a significant cooling effect with the increase of building height. For example, the LST rapidly decreases with the increase of building height within the range of 0~45m in Shanghai, 0~60m in Beijing, 0~51m in Chongqing, 0~45m in Guangzhou, 0~33m in Shenzhen, 0~36m in Tianjin, respectively. After the building height exceeds 66m, the impact of building height on LST is significantly weakened. It presents scattered points in Fig 6.

The reason why building height and LST has a negative correlation is discussed as followed.

**Fig 7:**
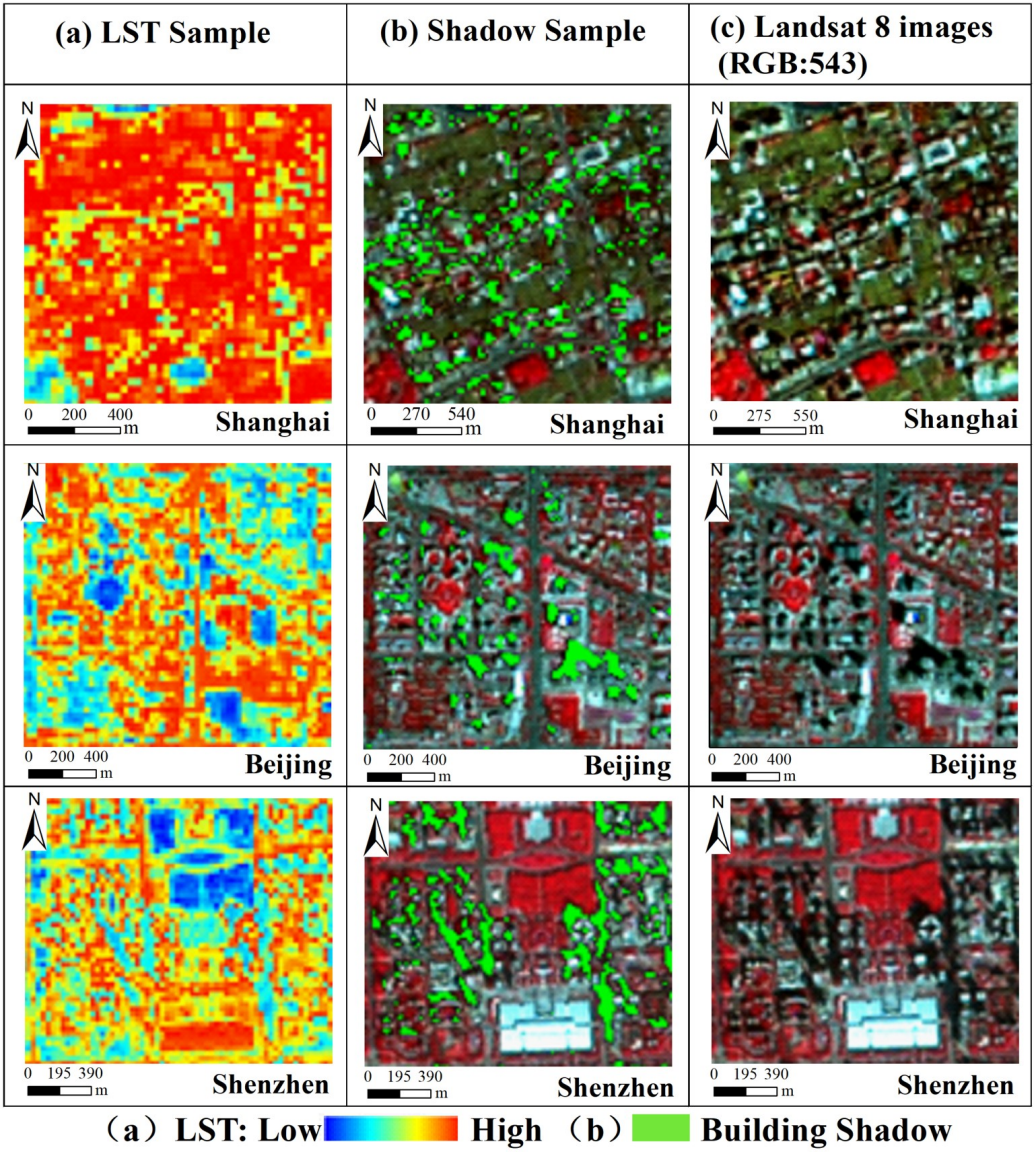
Relationship of spatial distribution between building shadow and LST (USGS/NASA Landsat 8).

1. In summer, building shade can cool urban temperature effectively by reducing total solar radiation in sunlight [38, 39]. Furthermore, nearby buildings could partly be relieved from the hot sunlight owing to building shadows during the day. We analyze the difference in LST by comparing the different building shadow samples in the six megacities. The results show that the shaded region is cooler than the surrounding non-shaded region (Fig 7).
2. Wind flow and air circulation help reduce the temperature [40, 41]. The existing buildings seldom set the ventilation hole in the central part. As a result, high-rise building area exists obviously wind break or wind-catching effect. It changed the original dominant wind direction around the high-building, which produces turbulence both in the windward and in leeward side. Furthermore, it makes "wind valley" in the local building region. This wind disturbance effect improves the local thermal environment in high-rise buildings area.
3. Urban architectural patterns form the different urban local climate zones (LCZs) and influence the change of LST [42–44]. The newly built-up areas mostly have a regular landscape structure with aligned and spare multi-storied and high-rise buildings LCZs, which facilitates air convection [45]. In contrast, the old district mostly has compact low-rise building LCZs. The urban street canyon increases the LST by trapping the heat inside compact structures and blocking the wind [46]. The LST of these LCZs is higher than those for open and spare building LCZs [43].
4. Since more horizontal active surface(the satellite nadir views on the ground) in low-rise building areas have been seen than that in the high-rise areas, these low-rise areas would appear disproportionally hot relative than high rise areas [47–49]. Moreover, owing partly to the lag in heating of large concrete masses, high-rise building areas appear cooler at this time of about 10:00 a.m. [47].

## Discussion and conclusion

### Discussion

The Municipal Planning Bureaus of the six megacities issued the relevant provisions of the planning and design notice, which stipulated the building height. For example, the building height in Beijing cannot exceed16 floors in the old center within the Second Ring Road. In Guangzhou, the building height cannot exceed 30m in the historical streets of Yuexiu District, Liwan District, and Haizhu District. It is necessary to strictly implement the relevant provisions and pay attention to the planning. In addition, it commits an error in the building height results which were all calculated by multiplying the number of floors by 3 m. Therefore, by referring to the online building height information of the six megacities and Google high-resolution imagery, the building height data in this paper has been corrected.

We focused on studying the relationships between building height and urban heat environment. In the future comprehensive urban heat environment analysis, it needs to consider many other factors, e.g., building materials, building density and vegetation. In addition, the correlation analysis used to analyzed the quantitatively relationship between LST and building height cannot well express their spatial correlation.

## Conclusion

Our study investigates the effects of the building height on the thermal environment in megacities. We employed bivariate correlation analysis to explore possible associations of building height with surface temperature, and achieved the following results.

1. The spatial distribution of high-rise buildings and super high-rise buildings are mainly concentrated in the center business districts, riverside zones, newly built-up area of the six megacities. In the urban area, the number and the building area ratio of the high-rise buildings (24-100m) and super high-rise buildings (>100m) account for over 5% and 4.74%, respectively. Among them, Chongqing’s urban area has the largest high-rise building number percent (18.26%) and building area ratio (19.37%).
2. Being the highly urbanized region, most of the urban areas in the six megacities are associated with high LST. Most of the high-temperature grid cells were distributed in industrial area and old center built-up area. The results show that Ninety-nine percent of the city areas of Shanghai, Beijing, Chongqing, Guangzhou, Shenzhen, and Tianjin are covered by the LST in the range of 30.2~67.8°C, 34.8~50.4°C, 25.3~48.3°C, 29.9~47.2°C, 27.4~43.4°C, and 33.0~48.0°C, respectively.
3. According to the regression analysis result, building height and LST have a negative logarithmic correlation with the correlation coefficient value ranging from -0.701 to -0.853. In the building’s height within 0–66m, LST will decreases significantly with the increase of building height. This indicates that the increase of building’s height will bring a notable cooling effect in this height range. After the building’s height exceeds 66m, its effect on LST will be greatly weakened. This is due to the influence of building shadow, local wind disturbances, and the layout of buildings.

## Supporting information

S1 File (RAR)
